# Meta-analysis of gene expression microarrays with missing replicates

**DOI:** 10.1186/1471-2105-12-84

**Published:** 2011-03-24

**Authors:** Fan Shi, Gad Abraham, Christopher Leckie, Izhak Haviv, Adam Kowalczyk

**Affiliations:** 1 National ICT Australia, Victoria Research Laboratory, Level 2, Building 193, The University of Melbourne Victoria 3010, Australia; 2 Department of Computer Science and Software Engineering, The University of Melbourne, Parkville, Victoria 3010, Australia; 3 Baker IDI Heart and Diabetes Institute, 250 Kooyong Road Caulield, Victoria 3162, Australia

## Abstract

**Background:**

Many different microarray experiments are publicly available today. It is natural to ask whether different experiments for the same phenotypic conditions can be combined using meta-analysis, in order to increase the overall sample size. However, some genes are not measured in all experiments, hence they cannot be included or their statistical significance cannot be appropriately estimated in traditional meta-analysis. Nonetheless, these genes, which we refer to as *incomplete genes*, may also be informative and useful.

**Results:**

We propose a meta-analysis framework, called "Incomplete Gene Meta-analysis", which can include incomplete genes by imputing the significance of missing replicates, and computing a meta-score for every gene across all datasets. We demonstrate that the incomplete genes are worthy of being included and our method is able to appropriately estimate their significance in two groups of experiments. We first apply the *Incomplete Gene Meta-analysis *and several comparable methods to five breast cancer datasets with an identical set of probes. We simulate incomplete genes by randomly removing a subset of probes from each dataset and demonstrate that our method consistently outperforms two other methods in terms of their false discovery rate. We also apply the methods to three gastric cancer datasets for the purpose of discriminating diffuse and intestinal subtypes.

**Conclusions:**

Meta-analysis is an effective approach that identifies more robust sets of differentially expressed genes from multiple studies. The incomplete genes that mainly arise from the use of different platforms may also have statistical and biological importance but are ignored or are not appropriately involved by previous studies. Our Incomplete Gene Meta-analysis is able to incorporate the incomplete genes by estimating their significance. The results on both breast and gastric cancer datasets suggest that the highly ranked genes and associated GO terms produced by our method are more significant and biologically meaningful according to the previous literature.

## Background

Gene expression microarrays are a high throughput technique for measuring gene expression levels in thousands of genes simultaneously, and have been widely used in the study of cancer genomics. An important application of gene expression microarrays is detecting differentially expressed genes by statistical analysis. For example, the classical *t*-test can be used to assess the statistical significance of genes in terms of their ability to discriminate samples from two phenotypes.

While many microarray experiments from different laboratories have been performed with the same research aim, the results of these experiments may differ from each other in many aspects, e.g., the platform, the probe sets or the characteristics of the samples. Consequently, the significant genes identified by the same statistical analysis from different experiments may be inconsistent.

To overcome these inconsistencies, the evidence from multiple studies needs to be combined. Several papers [1-3] directly integrated gene expression data by aligning genes/probes and concatenating samples. Meta-analysis [4] is another way of generating more robust and consistent statistical results by integrating multiple datasets and outputting an overall score, which we refer to as a *meta-score *for each gene/probe across all studies. For example, [5] integrated the p-values from the *t*-test, [6-8] integrated the effect size based on the model of [4], [9] integrated the ranks of genes, and [10] integrated the test statistics based on a mixture model of the normal distribution by considering the concordance between two datasets.

In addition, some papers used meta-analysis techniques to discover significant gene functions. For example, [11] applied meta-analysis directly to the functional categories associated with each individual dataset, rather than the expression data, in order to identify more significant pathways; [12] used meta-analysis to predict unknown functions of genes.

The integration of datasets from different platforms can generate more statistically significant results by reducing biases caused by specific platforms or experimental conditions. The study in [13] first highlighted the importance of the alignment between different platforms as an issue for the meta-analysis of gene expression microarrays. More recently, the studies in [1,2] applied meta-analysis to multiple platforms, and demonstrated that more robust gene signatures could be generated from multiple platforms.

**Figure 1:**
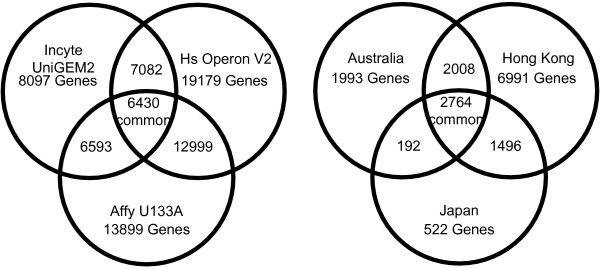
**Overlap between gene sets from different platforms**. The overlap between the gene sets from different microarray platforms. Left: Three platforms used in [14]. Right: Three gastric cancer datasets used in our experiments.

A challenge for meta-analysis in this context is that microarray datasets from different platforms do not usually possess an identical set of probes. Consequently, it is critical to fix a single set of probes as the candidates for statistical analysis. It is common to encounter incomplete alignment of genes among different microarray experiments, especially among those microarrays from different platforms. For example, the study in [14] compared three microarray platforms - one with short oligonucleotides, one with long oligonucleotides, and a cDNA platform. The three platforms have 6430 genes in common, but many more genes are shared by a pair of platforms or by a single platform, as shown in Figure 1. Similarly, many other meta-analysis studies have used datasets from different platforms, e.g. [5-7]. The overlap of genes among the three gastric cancer datasets [15-17] used in our experiments, which were independently generated by the research groups from Australia, Hong Kong and Japan on different platforms, is also shown in Figure 1. In addition, other reasons can also cause missing replicates in microarrays.

However, to the best of our knowledge, all existing methods of gene expression meta-analysis either only consider those features that are assayed in *all *datasets (which we refer to as *complete genes*), whereas the other genes that are not measured in all datasets are discarded, or simply ignore the missing replicates in the incomplete genes. We refer to the genes that are not measured in all datasets as *incomplete genes*.

However, the incomplete genes may also be significant and should be considered as candidates, even though their significance is not tested in all studies. In this paper, we focus on developing a novel meta-analysis method that takes complete and incomplete genes into account simultaneously.

We propose a meta-analysis framework, called *Incomplete Gene Meta-analysis *(IGM), which is able to incorporate incomplete genes caused by cross-platform integration or any other reasons for missing replicates. IGM comprises three major steps: (1) Compute a statistic for every replicate (each probe in each dataset) using the Hedges' *g *effect size [4]; (2) Impute the significance of *missing replicates*, where the incomplete genes are not measured in particular datasets, using the model of a conditional probability distribution over the datasets; (3) Generate an overall significance score (meta-score) for each probe across all datasets using a variant of an earlier linear model [4,6,18]. As a basis for comparison, we also implemented other variants of this framework by replacing its key steps, including a traditional approach that does not consider the incomplete genes and a method that simply ignores the missing replicates in the incomplete genes.

We first tested IGM and the comparable approaches on five breast cancer datasets with an identical set of probes, for the purpose of distinguishing the binary label of a given number of years to metastasis. We simulated the incomplete genes by randomly removing a subset of probes from each dataset. A gene ranking was generated using each method and the false discovery rate (FDR, [19]) was estimated using a permutation test [6,20]). Our method consistently achieved the closest FDR to that of the gene ranking produced on the original datasets without incomplete genes, which was considered as the gold standard. We also conducted experiments on three gastric cancer datasets, which were generated independently by research institutions in Australia [15], Hong Kong [16] and Japan [17], for the purpose of discriminating diffuse and intestinal subtypes of gastric cancer [21]. Using an enrichment test for Gene Ontology terms in both groups of cancer datasets, IGM identified more significant terms that were closely related to a particular subtype of gastric cancer than only using complete genes. The above results show that the highly ranked genes produced by IGM were statistically and biologically more significant than those produced by the other methods.

In Section, we describe the IGM framework, the comparable methods and our evaluation metrics. In Section, we present the experimental results on the breast cancer and gastric cancer datasets. In Section, we discuss the biological relevance of the results on the gastric cancer datasets. Finally, we conclude the paper in Section.

## Methods

In this section, we describe our framework called Incomplete Gene Meta-analysis (IGM), which incorporates both complete genes and incomplete genes simultaneously by including the key step of imputing the significance of missing replicates. We also propose several other variants of this framework as a basis for comparison using three types of evaluation metrics.

### Notation

Before presenting our framework, we first introduce several concepts and notations that are used in the following sections. We are given *k*(*k *≥ 2) gene expression datasets *GE_j _*= (*G_j_*, *S_j_*), *j *= 1, ···, *k*, where the dataset *GE_j _*comprises the gene set *G_j _*and the sample set *S_j_*. Let *G_I _*and *G_U _*denote the intersection(1)

and union(2)

of all gene sets, respectively. If the gene *g_i _*∈ *G_U _*is not measured in the dataset *GE_j_*, *j *∈ { 1, ···, *k*}, we call it a *missing replicate*. A gene that has no missing replicates is called a *complete gene*. Otherwise, it is called an *incomplete gene*.

Note that the features are aligned by their gene symbols between datasets. While there are other strategies to align probes between studies, they are not the focus of this paper. More details about the alignment can be found in [22].

If multiple probes in one dataset correspond to a single gene, the median expression level of these probes is computed for each sample.

### Incomplete Gene Meta-analysis Framework

**Figure 2:**
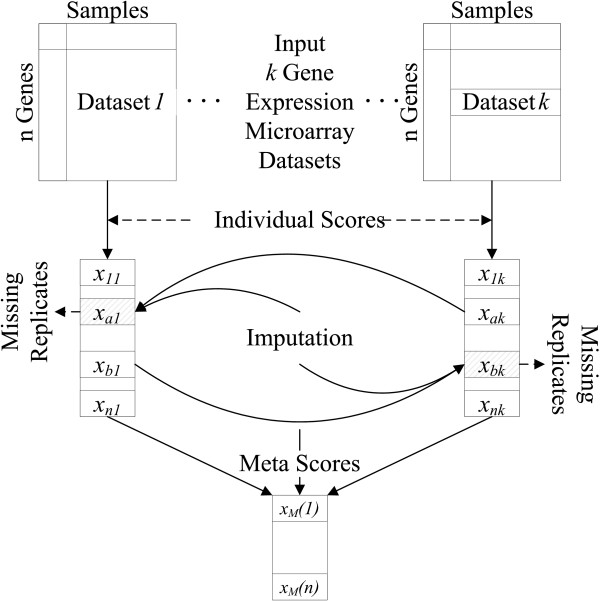
**Incomplete Gene Meta-analysis**. The process of Incomplete Gene Meta-analysis.

Our Incomplete Gene Meta-analysis framework computes an overall score, called a *meta-score*, for each gene across all datasets, by imputing the significance of missing replicates and integrating the statistical results from individual datasets. The major steps are as follows (see Figure 2).

1. **Input **- We are given *k *≥ 2 gene expression microarray datasets *GE_j _*= (*G_j_*, *S_j_*), *j *= 1, ···, *k*. In each dataset, the samples are labeled with different phenotypes or clinical annotations, with respect to which the differentially expressed genes can be detected.

2. **Candidate gene set **- We have to select a candidate gene set *G*_0 _⊆ *G_U _*if the gene sets differ between datasets. Previous methods (e.g., [6,9,10]) only select complete genes (*G*_0 _= *G_I _*), but we select *G*_0 _= *G_U _*, so that all genes are considered as candidates. Let *n *= |*G*_0_| denote the total number of candidate genes.

3. **Individual scores **- We apply a statistical test to each replicate *g_i _*in dataset *j*, so that a score *x_ij_*, which could be the test statistic or p-value, is used to measure the significance of the replicate. We let(3)

denote the score matrix for all *n *genes in *k *datasets. The corresponding value of any missing replicate is initially undefined.

4. **Imputation **- For each missing replicate, we impute a value  for *x_ij _*so that it has a valid score. We estimate the scores of the missing replicates using a probability distribution that is conditional on the observable replicates, and also calculate the estimation error for the imputed scores.

5. **Meta-scores **- We compute a meta-score *x_M _*(*i*) for every gene *g_i_*, characterising its overall significance across all datasets.

In the following three subsections, we discuss the details of steps 3 to 5.

### Individual Scores

Many statistical tests could be used for measuring the significance of the differential expressions of genes. In the case of two phenotypes, we employ the Hedges' *g *effect size [4], which is defined as the standardized difference in the means between two populations. We first briefly describe the general case of estimating the Hedges' *g *effect size from the two groups of samples for one gene in one dataset. A biased estimator is given by:(4)

where *ē*_1 _and *ē*_2 _are the mean values of the samples in groups 1 and 2, respectively, and s* is the pooled standard deviation of the samples. Using *g *in Equation (4), an unbiased estimator g* of the population effect size is given by:(5)

In addition, the variance of g* can be estimated using:(6)

where *n*_1 _and *n*_2 _are the numbers of samples in groups 1 and 2, respectively.

In our algorithm, we compute g* as the individual score (Section) for each observable replicate *g_i _*in dataset *j*:(7)

while the score for each missing replicate is initially undefined.

### A Variant of the Linear Model for Meta-scores

Our problem corresponds to the general problem of estimating the population effect size from a given set of measurements. We first recall an existing method for estimating a population parameter used by [4,6,18]. The observed statistic *x_ij _*in Equation (7) for the replicate *g_i _*in dataset *j *is hierarchically modeled as follows:(8)(9)

In this model, *μ_i _*is the unknown population effect size to be estimated for gene *i*. A key challenge in this estimation problem is how to account for the variation within each study (modeled by *β_ij_*) as well as the variation between studies (modeled by *α_ij_*). We now consider each of these terms.

First, many factors, such as different microarray platforms or samples of different ages and regions, may affect the measurements and result in variations of the population effect size between studies. This is modeled by the error term *α_ij _*in Equation (9), which follows a normal distribution with 0-*mean *and . The term *μ_ij _*is the study-specific population effect size.

Second, the other error term *β_ij _*in Equation (8) represents the variation in measuring *μ_ij _*due to the finite number of samples in each study. This term's variance  is estimated by Equation (6).

An unbiased estimator of *μ_i _*is given by the semi-weighted mean [4,6,18]:(10)(11)

where  and  are estimates of the population parameters  in Equation (9) and  in Equation (8), respectively.

When there is no variation between studies, which indicates , every study has the identical population effect size *μ_ij _*= *μ_i_*. In this case, the model is called a *Fixed-Effects Model *(FEM). Otherwise, the model is called a *Random-Effects Model *(REM), in which . The test for FEM or REM and the estimate of  in Equation (9) can be found in [4,6,18,23].

Thus, when incomplete genes are absent, we can directly use this estimate as the meta-score:(12)

To incorporate the imputation step described in Section, we propose a variant of the above model. In our case, some of the *x_ij _*are unobservable. As a consequence, the imputation of the scores for these missing replicates ( in Section) will lead to an additional source of variation, which can be accounted for by introducing a new error term *e_ij _*in the model:(13)

For all observable replicates, *e_ij _*= 0. This indicates that the new error term is only introduced for the missing replicates. We extend the semi-weighted mean in Equation (10) to a form involving *e_ij _*as follows:(14)(15)

where  is the estimated variance of *e_ij_*, which is determined by a specialized method of imputation. Thus, when incomplete genes are present, we use this estimate  of the semi-weighted mean as the meta-score:(16)

We can explain the impact of the error term *e_ij _*as follows. First, if the expectation of the error term *E*(*e_ij_*) = 0, which implies the estimate of the score of a missing replicate in Equation (13) is unbiased:(17)

The estimate  in Equation (14) is again an unbiased estimate of *μ_i_*. Otherwise,  could overestimate or underestimate *μ_i_*. depending on the method of imputation. Second, intuitively, the imputed scores will have a smaller weight  in Equation (15), due to the inclusion of the estimated variance  of the new error term.

### Imputation using Conditional Probability

The imputation step enables the incomplete genes, which are usually neglected in previous studies, to be included in the meta-analysis.

We use a conditional probability distribution (CPD) for imputation. When detecting differentially expressed genes in multiple datasets with respect to the same type of sample labels (e.g., tumor vs. normal), the scores between datasets are usually positively correlated, which reflects the consistency between datasets in terms of significant genes. Otherwise, the meta-analysis is pointless. Intuitively, a gene that is observed to be differentially expressed in most studies is also expected to be significant in the studies where the gene is missing. Based on this, we can estimate the unobservable scores conditioned on the observable scores of the same gene in other studies.

#### 1. *Distribution model*

For the score matrix *X *= [*x*_*ij*_]_*n *× *k *_in Equation (3), we denote **x_i_**., *i *= 1,···, *n*, as the vector of the *i*th row (feature), and **x**.**_j_**, *j *= 1,···, *k*, as the vector of the *j*th column (dataset).

In our model, the row vector **x_i_**. follows a *k*-dimensional normal distribution across *k *datasets:(18)

where the dimensions (columns **x._j_**) are usually positively correlated.

We denote **p_i _**⊂ {1, ···, *k*} as the set of indices of the unobservable dimensions (missing replicates), and **q_i _**⊂ {1, ···, *k*} as the set of the observable dimensions, so that |**p_i_**| + |**q_i_| **= *k *and **p_i _**∩ **q_i _**= Ø. For gene *i*, the distribution of the unobservable sub-vector  conditional on the observable sub-vector  is given by:(19)(20)(21)

where(22)

More details of the conditional multivariate normal distribution can be found in [24]. Note that the approximate normality of the real datasets used in our experiments is shown in the Additional File 1.

#### 2. *Parameter estimation*

The above parameters *μ *and **Σ **are computed from all complete genes using maximum likelihood estimation. Consequently, we can obtain the conditional probability distribution in Equation (19).

#### 3. *Imputation*

Given the CPD in Equation (19), the most likely score for the missing replicates is given by the mean of the distribution. Thus, the score  for missing replicate *g_ij _*in Equation (13) is imputed as an element of the sub-vector:(23)

where  is computed in Equation (20).

However, the CPD allows other possible values for estimating the scores of missing replicates, which leads to the variation of imputation. The variance of this estimate, which is modeled by the error term e*_ij _*in Equation (13), is given by the diagonal elements of the covariance matrix  in (21) of the CPD:(24)

where  is computed in Equation (21).

Consequently, the imputed scores  for missing replicates in Equations (13) and (14) and the estimated variance of imputation  in Equations (13) and (15) can be obtained using our strategy, and are used to compute the meta-scores.

In summary, the intuition of the CPD strategy is to impute the scores of missing replicates based on the positive correlation between datasets, which is also the basis of meta-analysis. We discuss the reasons why we employ such an imputation strategy here.

1. Choice of distribution: Assuming a multivariate normal distribution for data is a typical way to estimate missing values in incomplete data, even if the real distribution is not exactly normal [25]. The multivariate normal assumption enables the use of a tractable conditional probability model and captures the correlation between datasets, which is usually present and positive when we apply statistical tests to multiple datasets with respect to the same type of clinical annotation.

2. Unbiased estimation: Under the proposed model, the imputation provides an unbiased estimate of the scores for missing replicates (Equation (23)), which is desirable for an accurate estimate of the population effect size (*E*(*e_ij_*) = 0 in Section).

3. Variation of imputation: A critical aspect of imputation is how to model the instability of estimating missing values, which is reflected as the variance of imputation (Equation (24)). In the survey of [26], two types of imputation, "model-based imputation" [25,27] and "multiple-imputation" [28] dealt with this problem by using the EM algorithm and estimating multiple values for missing entries, respectively. However, since our model itself provides an estimate of the imputation variance based on the CPD, this variance can thus be directly used in the linear model in Equation (13). This strategy, which includes the variance of imputation as part of the model, avoids the iterative procedure in the EM algorithm, which can be costly for large-scale studies. Moreover, it also avoids repeatedly applying the downstream analysis to the multiple versions of imputed datasets that would arise in multiple imputation. Overall, our imputation is considered to be a "composite method" comprising "model-based imputation" and "cold deck imputation" [26] with a strategy of embedding the variance in the meta-analysis model.

However, the CPD model has a potential limitation due to the assumption of the multi-normal distribution in Equation (18). In this assumption, the effect sizes of all genes follow a multi-normal distribution with the same mean (*μ*). This assumption may not always hold because the effect sizes of differentially and non-differentially expressed genes may come from different distributions. On one hand, the number of differentially expressed genes is relatively small in practice, and we demonstrate its validity for imputing incomplete genes in Section 3. On the other hand, this issue has been considered in [10], where a mixture model was proposed for differentially and non-differentially expressed genes. Thus, the integration of a mixture model for refining the imputation stage will be investigated in our future work.

Another potential limitation of this imputation method is the lack of modeling of the dependence between studies when estimating the true effect size in Equation (14). Although this model has assigned a smaller weight to the imputed effect sizes in order to compensate the variability of imputation, the dependence caused by the CPD in Equation (19) has not been taken into account. A topic for future research is to establish a model that incorporates this inter-study dependence.

### Comparable Methods

In addition to the algorithm described above, we have also implemented several other methods to evaluate the importance of including incomplete genes and properly imputing their significance. The Hedges' g effect size [4] is used in all methods to compute the individual scores, and the model described in Section is used to compute the meta-scores. The comparable methods that we have implemented are as follows.

1. INTERSECTION: All incomplete genes are discarded as in earlier meta-analysis methods. Thus, the candidate gene set *G*_0 _is the intersection of the gene sets in all datasets (*G_I_*). The imputation step is not necessary. In this case, IGM is equivalent to the method of [6].

2. IGNORE: Both complete genes and incomplete genes are taken into account, by simply ignoring the missing replicates in the incomplete genes. Meta-scores are computed based only on the observable replicates in the incomplete genes. A typical example of this type of method can be found in [29].

These comparable methods are designed for different purposes. By comparing with the INTERSECTION method, we can show the importance of including incomplete genes. The Ignore method is also considered because it is the simplest way of incorporating incomplete genes.

### Evaluation Metrics

In order to evaluate the statistical significance of the differential expression of genes, we use the false discovery rate estimated by the permutation test [6,20] as our metric. We also use the Gene Ontology [30] to assess the significance of the biological processes that are enriched in the significant genes identified by our methods. In the Additional File 1 we also consider the effect of incomplete genes on classification accuracy.

#### False Discovery Rate

The false discovery rate [19] is defined as the ratio of the number of false positives to the number of features declared significant according to a specific ranking of features. However, when the gold standard for the true positives is not available, the FDR is usually estimated from the data. In our experiments, we employed the permutation test used by [20] and [6] to estimate the FDR.

The idea behind this method [6,20] is to estimate the number of false positives at a given significance level by randomly permuting the labels of samples. We assume that we need to estimate the FDR at the significance level of *x_M _*(*i*), which is the meta-score of *g_i _*and is ranked *R_i _*from the most to least significant. In the *b*th permutation, the labels of samples are independently permuted in every dataset, ensuring that the number of samples in each class is unchanged. We then repeat the process of meta-analysis, and produce a vector of meta-scores  in this permutation. For the unpermuted meta-score *x_M _*(*i*) associated with *g_i_*, the number of false positives is estimated as the number of permuted meta-scores greater than or equal to *x_M _*(*i*) in this permutation. After a total number of *B *permutations, the Expected number of False Positives (EFP) is computed as the average number of false positives across all permutations. Consequently, the FDR at *x_M _*(*i*) is the ratio of EFP to the number of genes declared significant at the threshold of *x_M _*(*i*), which is the rank *R_i_*.(25)(26)

#### Gene Ontology Significance

To assess the ability to identify significantly over-represented GO terms, we compute the significance of GO terms associated with each subset of significant genes ranked by our methods. A p-value is computed for each GO term using Fisher's exact test, where a small p-value implies that this term is significantly over-represented. In our experiments, we only consider the Gene Odontology branch "Biological Process."

## Results

In this section, we first summarise the IGM algorithm whose details are described in Section. We then apply the IGM algorithm as well as the other approaches in Section to three separate sets of gene expression microarrays: five breast cancer datasets generated on the same platform, three gastric cancer datasets from different platforms and eleven different types of cancer datasets from the same platform. By comparing their performance in terms of the false discovery rate and the Gene Ontology terms, we show that compared with the other approaches IGM is more able to identify significant genes and GO terms that have been proven to be closely related to these cancers by the previous literature.

While our aim is to support meta-analysis across different microarray platforms, we first need to test the accuracy of our approach under controlled conditions. We achieve this in Section by analysing five breast cancer datasets from the same platform, where we can simulate incomplete genes by randomly removing genes from each dataset. In this way, we can validate the accuracy our method by comparing the results of meta-analysis with and without the incomplete genes. Having evaluated the accuracy of our approach under controlled conditions, we then evaluate its performance on three gastric cancer datasets that were generated on different platforms in Section. Finally, we test our method on a larger scale of 11 cancer datasets.

### IGM Algorithm

We summarise the key steps of the IGM algorithm as follows.

1. Input - *k *(*k *≥ 2) gene expression microarray datasets *GE_j _*= (*G_j_*, *S_j_*), *j *= 1, ···, *k*.

2. Alignment - Calculate the union set of features in all studies , *n *= |*G_U_*|

3. Effect sizes - Compute the effect size *x_ij _*of each feature *i *in study *j *for all features in *G_U _*.

4. Imputation - Impute the statistic of the missing replicates in the above score matrix *X *using the CPD method in Section. The scores matrix with imputed significance is denoted as:

5. Meta-score - Compute the meta-scores *x_M _*(*i*) for all features based on the score matrix *X' *using the model in Section.

In our implementation, we have also provided an option to filter out the features with only a small proportion (e.g., 30%) of observable replicates in order to avoid unstable imputation.

In addition, we also implemented the INTERSECTION and IGNORE methods in Section by specifying different options in the framework in Section. These two methods are the basis of comparison with our method in the evaluation. The main IGM program was implemented in Matlab and the source code is provided in the Additional File 2.

### Controlled Evaluation of Accuracy in Breast Cancer Datasets

As a first step, we need to evaluate the accuracy of our IGM. However, this raises the question of how to measure accuracy in the absence of any ground truth of the significance of each gene, especially for incomplete genes. In order to generate such a ground truth for a controlled evaluation, we have simulated missing replicates in five breast cancer datasets from the same platform. In this way, we can compare the accuracy of the meta-scores generated for each gene with simulated missing replicate(s), by making a comparison with the meta-scores generated where all replicates are present in the original datasets. The meta-scores from the original datasets with no missing replicates thus become a "gold standard" for our evaluation, since using more samples leads to more reliable results. The results of our evaluation are presented in Section and.

#### Breast Cancer Datasets

We used five public breast cancer datasets from NCBI GEO [31]: GSE2034 [32], GSE4922 [33], GSE6532 [34,35], GSE7390 [36], and GSE11121 [37], all on the Affymetrix HG-U133A platform. The phenotype was a binary label (< 5, ≥ 5) years to metastasis.

#### Simulating Missing Replicates

Assuming that the probes are missing in each dataset independently, we randomly removed a proportion of probes (30% in the following experiments) from each dataset to simulate missing replicates. We then tested each meta-analysis approach on these datasets with simulated missing replicates. Subsequently, by comparing the results with the gold standard (the gene ranking generated on the original datasets), we can evaluate the ability of the approach to estimate the significance of incomplete genes.

#### FDR Comparison

**Figure 3:**
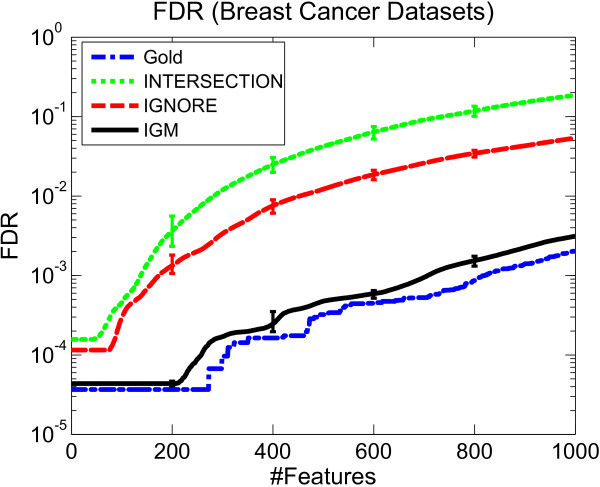
**FDR evaluation on breast cancer datasets**. The average FDR of different meta-analysis methods in the breast cancer datasets. Except for the FDR computed on the original datasets which is used as the gold standard (labeled "Gold"), the other methods were averaged across 100 groups of datasets with simulated missing replicates. The 95% quantiles of the FDR across the 100 simulations are given by the error bars at the number of features 200, 400, 600 and 800.

In this section, by comparing the FDR between different methods, we demonstrate that IGM is able to better estimate the significance of incomplete genes than the INTERSECTION and IGNORE methods. We first applied the framework in Section to the original five datasets without any missing replicates (hence, imputation is not necessary) to generate a gene ranking, and computed the FDR using the permutation method in Section as the gold standard for comparison. In this case, IGM is equivalent to the method in [6]. We then generated 100 groups of datasets with simulated missing replicates using the approach described in Section. For each group of datasets, we generated a ranking of all probes using IGM as well as the other methods, and computed the average FDR across the 100 groups of datasets for each method. The resulting FDR (in log scale) versus the number of probes declared significant for the "gold standard", IGM and the comparable methods, are shown in Figure 3. In addition, the 5% and 95% quantiles of the FDR across all 100 simulations are shown at several positions to demonstrate the significance of the differences between these methods.

In our comparison, we consider that the probe ranking generated on the original datasets without any missing replicates, where most information is available, is most reliable, and we refer to this as our "gold standard". Note that the FDR for the gold standard is non-zero because some genes in the original dataset are significant just by chance.

All methods when applied to the datasets with simulated missing replicates produce the same results for *complete genes*; the difference between these methods is reflected in their ability to estimate the significance *of incomplete genes*.

We analyse the cause of the overestimation of the FDR as follows. If some incomplete genes are often assigned less significant scores by a particular method than the significance level that they should have in the gold standard, these genes have a greater chance to be counted as false positives (see Section for details). In this case, the FDR is likely to be overestimated due to the increased number of false positives. For example, in Figure 3 since the INTERSECTION method discarded all incomplete genes, which is equivalent to assigning the least significant score (e.g., p-value = 1) to them, the FDR is overestimated compared to the gold standard. In the Ignore method, the estimated significance of incomplete genes is merely determined by the observable replicates and the inter-study correlation is neglected. Thus, the estimated significance is likely to be distorted by those observable values, and so the estimated FDR deviates from the "gold standard".

Thus, we aim to develop a meta-analysis method that generates an FDR as close as possible to the FDR generated by the gold standard, indicating that this method is able to precisely estimate the significance of probes even though some replicates are missing. In this regard, our approach outperforms the others, since it is closest to the gold standard, and the significance of this difference in the FDR distributions is demonstrated by Figure 3.

#### Gene Ontology Terms

To further compare the ability of each method to find a more significant set of genes, we have also evaluated the GO terms found in the five breast cancer datasets.

In this experiment, we used the probe rankings produced by the gold standard, INTERSECTION and IGM, which are identical to the results in Section. Based on these rankings, a subset of significant probes (FDR≤0.01) were selected for each method and each simulation of missing replicates. To assess the significance of enrichment, we used Fisher's exact test to compute the p-values of GO enrichment in these significant subsets. The Biological Process branch was used. Subsequently, for the INTERSECTION and IGM methods, we computed the geometric mean of the p-values of the GO terms from all 100 simulations, in order to generate a single integrated list of GO terms as a basis for comparison with the terms produced by the gold standard. In Table 1 the top four GO terms for each method are listed.

**Table 1:** Top GO Terms in breast cancer datasets

gold standard (87 terms)		IGM (60 terms)		INTERSECTION (2 terms)		IGNORE (29 terms)	
**GO Term**	**p-value**	**GO Term**	**p-value**	**GO Term**	**p-value**		

phosphoinositide-mediated signaling	3.87E-14	phosphoinositidemediated signaling	1.24E-13	phosphoinositide mediated signaling	2.70E-03	phosphoinositidemediated signaling	2.44E-11

mitotic chromosome condensation	5.61E-13	mitotic chromosome condensation	2.14E-11	mitotic chromosome condensation	5.34E-03	mitotic chromosome condensation	1.47E-08

DNA replication	1.22E-08	spindle organization	1.48E-08	regulation of cyclin-dependent protein kinase activity	1.33E-02	spindle organization	1.91E-08

spindle organization	1.53E-08	DNA replication	1.53E-08	DNA repair	1.47E-02	DNA replication	4.13E-06

Top four GO terms that are over-represented in the set of significant probes generated by the gold standard, IGM, INTERSECTION and IGNORE methods in the breast cancer datasets.

As with the FDR evaluation, a good meta-analysis method is expected to reproduce the order of GO terms generated by the gold standard as much as possible when missing replicates are present. Before comparing the INTERSECTION and IGM with the gold standard, we first show that the gold standard has effectively identified the important GO terms associated with the time to metastasis of breast cancer.

A short time to metastasis (less than five years) has been linked to up-regulation of the genes related to cell cycle, cell proliferation, and cell invasion [32,38]. The significant GO terms generated by the gold standard confirm that the up-regulation of the biological processes related to cell cycle, such as mitotic chromosome condensation, spindle organization, DNA replication and DNA repair [32,38-40], the processes related to signal transduction, such as phosphoinositide-mediated signaling [32,38], and cell proliferation [40] are most strongly associated with the short time to metastasis.

**Figure 4:**
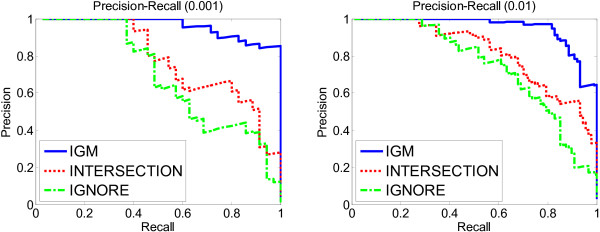
**Precision-recall of GO terms**. Precision-recall curves of GO terms in the breast cancer datasets. Left: the true significant terms are annotated from the gold standard under the threshold 0.001. Right: the true significant terms are annotated from the gold standard under the threshold 0.01.

In order to statistically show the advantages of IGM, we compared the precision and recall of the INTERSECTION and IGM methods in identifying the significant GO terms found by the gold standard. First, in order to establish a gold standard for comparing GO terms, we selected the true significant GO terms from the gold standard method by setting a threshold α on the *p*-values. For example, given *α *= 0.01, we may find a set of GO terms in the gold standard with a *p*-value ≤ *α*, and denote this set as *G*. Second, we ordered all GO terms in the other methods, including the IGM, INTERSECTION and Ignore methods according to their *p*-values separately. Third, for each method (IGM or INTERSECTION), we scanned the ordered GO terms from the most significant to the least significant, and declared different numbers (top *k*) of GO terms as significant terms (where *k *ranges from 1 to all GO terms). Finally, for each number of terms declared significant *k*, we compared these terms declared significant with the true significant terms in the set *G*, which was previously obtained from the gold standard, and computed the precision and recall for this *k*. Thus, we can generate a vector of precision-recall pairs for different values of *k *as a curve shown in Figure 4. This procedure is similar to the generation of a ROC curve.(27)

Figure 4 shows the precision-recall curves across the ranked terms in each method, generated under the threshold *α *= 0.001 and *α *= 0.01. The higher precision and recall of IGM demonstrate that IGM better reproduced the order of GO terms in the gold standard than the INTERSECTION method.

**Figure 5:**
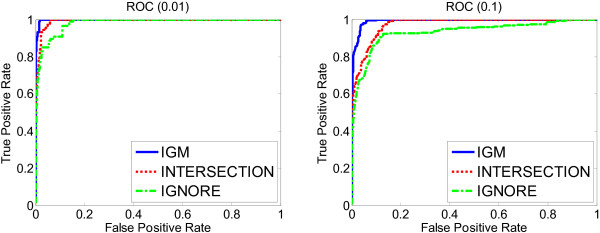
**ROC of GO terms**. ROC curves of GO terms in the breast cancer datasets. Left: the true significant terms are annotated from the gold standard under the threshold 0.01. Right: the true significant terms are annotated from the gold standard under the threshold 0.1.

Similarly, the ROC curves of GO terms can be also generated for different thresholds. We show the comparison of ROC curves between the IGM, INTERSECTION and IGNORE methods in Figure 5. The results confirmed that our IGM method was closest to the gold standard in terms of reproducing the significant GO terms.

**Figure 6:**
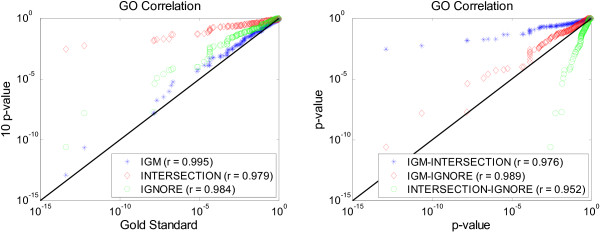
**Agreement of GO terms**. Scatter plot of all GO terms between different methods in the breast cancer datasets. Left: the agreement between the IGM, INTERSECTION, IGNORE methods and the gold standard. Right: the agreement between the IGM, INTERSECTION, IGNORE methods.

We have computed the correlation coefficients of the GO terms between the IGM, INTERSECTION and IGNORE methods and the gold standard, and the result in the form of a scatter plot is shown in Figure 6. The left figure shows the scatter plot of all GO terms between the three methods and the gold standard. Our IGM method reproduced the GO terms and their significance from the gold standard better than the other two methods, because it achieved the largest agreement with the gold standard (closest to the ideal diagonal line and the highest correlation coefficient). In addition, we also computed the agreement of the GO terms between the IGM, INTERSECTION and IGNORE methods in the right figure. The full list of these ranked GO terms for all methods is provided in the Additional File 3.

### Real Missing Replicates in Gastric Cancer Datasets

#### Gastric Cancer Datasets

We tested our IGM algorithm on three gastric cancer datasets, which we refer to as the Australian dataset [15] (6957 genes), the Hong Kong dataset [16] (13; 258 genes) and the Japanese dataset [17] (4974 genes). These three datasets were generated on different spotted cDNA platforms and do not possess an identical set of probes. We aligned the features by their gene symbols. Since we focused on the signatures discriminating two well-known subtypes of gastric cancer, diffuse and intestinal, according to Lauren's classification [21], only the tumor samples were retained. The Australian dataset has 35 diffuse samples and 22 intestinal samples, the Hong Kong dataset has 13 diffuse samples and 68 intestinal samples, and the Japanese dataset has 5 diffuse samples and 17 intestinal samples.

#### Gene Ontology Terms

We evaluated the significance of GO terms enriched in the top ranked genes in the gastric cancer datasets. We applied all methods to all three gastric cancer datasets, and set a threshold of FDR ≤ 0.01 to produce a subset of significant genes based on the resulting ranks using each method. The FDR was estimated using the approach in Section. We used GOstat [41] to detect the enriched GO terms for each subset of significant genes and to generate the corresponding FDR-corrected p-values [19]. In Table 2 we show the top GO terms over-represented in the groups of significant genes. Note that the significant genes were divided into two groups, which are prominently over-expressed in the diffuse and intestinal subtypes, respectively.

**Table 2:** Top GO terms in gastric cancer datasets

IGM		INTERSECTION		IGNORE	
**GO Term**	**p-value**	**GO Term**	**p-value**	**GO Term**	**p-value**

**Diffuse**					

DNA metabolic process	0	regulation of mitosis	7.80E-05	regulation of progression through cell cycle	5.83E-07

cell division	0	mitotic cell cycle	1.04E-03	regulation of cell cycle	5.83E-07

cell cycle	0	mitosis	1.22E-03	regulation of mitosis	9.78E-07

mitotic cell cycle	0	mitotic cell cycle checkpoint	1.22E-03	response to endogenous stimulus	1.11E-06

Intestinal					

biological adhesion;	0	muscle contraction;	3.40E-05	biological adhesion	3.13E-07

cell adhesion;	0	muscle system process;	3.40E-05	cell adhesion	3.13E-07

muscle development;	0	muscle development;	1.35E-03	multicellular organismal process	1.35E-05

muscle contraction;	2.80E-04	multicellular organismal process;	4.84E-03	muscle contraction	1.61E-05

Top four GO terms over-represented in the subset of genes that are prominently over-expressed in diffuse(first four terms) and intestinal (second four terms) subtypes of gastric cancer.

Since a few incomplete genes were included in the significant set and participated in some biological processes closely associated with a particular subtype of gastric cancer, such as "biological adhesion" enriched in the diffuse subtype (Table 2), the genes identified by IGM resulted in more over-represented terms that have been validated to be related to these subtypes in the previous literature (discussed in Section) than the INTERSECTION method. Under a threshold of the corrected p-value ≤ 0.01, IGM resulted in 73 significant terms while the Intersection method resulted in only 20 significant terms. This result is consistent with what we observed in the breast cancer datasets.

### A Validation on 11 Cancer Datasets

In order to validate the empirical performance on a larger number of studies, we have applied our method and the Intersection, Ignore methods to a group of 11 datasets with different types of cancer with the purpose of discriminating normal and cancer samples. A similar application can be also found in [2]. These datasets are all publicly available in GEO [31] (GEO series numbers are GSE781, GSE2719, GSE3868, GSE7670, GSE9476, GSE9750, GSE14359, GSE15852, GSE19147, GSE22529 and GSE23400).

**Figure 7:**
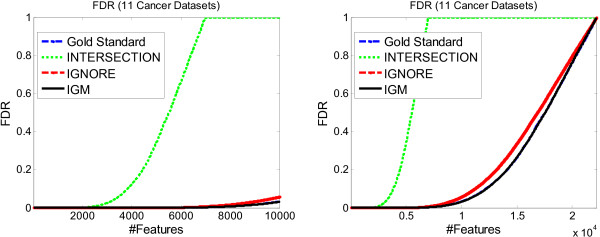
**FDR evaluation on 11 cancer datasets**. The average FDR of different meta-analysis methods in the 11 cancer datasets. The same experimental settings were used as the five breast cancer datasets.

All 11 datasets were selected on the Affymetrix HG-U133A platform in order to conduct the same evaluation as for the breast cancer datasets. We used identical settings with the experiments of the five breast cancer datasets except that the proportion of missing values in each dataset was set to 10% instead of 30% in order to retain enough features for the Intersection method. The FDR comparison for all the methods is shown in Figure 7.

As shown in Figure 7 our IGM method still performs better than the Intersection and Ignore methods in terms of FDR, since it is closest to the gold standard in the entire range. However, the performance of IGM is closer to the Ignore method than the result for the breast cancer datasets (Note that the left figure in Figure 7 shows the FDR for the top 10,000 features, while Figure 3 shows the FDR for the top 1000 features only. This is because the difference between different methods is too small for selecting a small number of features).

Due to the noise and inconsistency when the number of studies increases, the inter-study correlation may decrease. As a result, the imputation based on the inter-study correlation may not be as effective as the situation where a significant positive inter-study correlation exists (as with the breast cancer datasets).

Thus, this might be a reason for the reduced difference between our IGM method and the *Ignore *method. A previous study [10] considered the inter-study concordance in order to assess whether these studies are worthy of being integrated. Thus, as future work, we may take into account the inter-study concordance into the imputation step of our algorithm in order to improve the performance in large scale studies.

## Discussion

Here we discuss the biological relevance of the genes and GO terms that are over-expressed in the diffuse and intestinal subtypes separately.

Compared to intestinal gastric cancer, the most significant feature of the diffuse subtype is the poor differentiation caused by the invasion of tumor cells to the stroma [15,21,42].

The term "extracellular structure organization and biogenesis" and its descendent term, "extracellular matrix organization and biogenesis", which are associated with an important component of tumor invasion and metastasis, the extracellular matrix (ECM) [43,44], were over-represented in our experiment. In these terms, aside from the genes COL4A6, COL6A2 and COL14A1 belonging to the collagen family, Tenascin-X (TNXB), which was described as a metastasis signature in breast cancer [45], was also up-regulated in our experiment but has not previously been reported for gastric cancer. This is a potentially new discovery and provides a focus for further investigation.

Another feature of the diffuse subtype, active cell mobility, e.g., over-expression of Caldesmon 1 (CALD1), stimulates the invasion and metastasis of tumor cells [17,44]. This was reflected by the over-representation of the term "cell mobility" and its parent "localization of cell" in our experiment.

A few genes, such as the receptor tyrosine-protein kinase erbB-3 (ERBB3), which is related to growth factors [17], and dual specificity protein kinase (TTK) [46], which is related to cell proliferation, were found to be up-regulated in the intestinal gastric cancer samples. The over-expression of these features were reflected by the over-representation of several terms related to "cell cycle", such as "mitotic cell cycle" and "M phase of miotic cell cycle".

By analysing the statistically significant terms and their biological relevance, we observe that the gene sets identified by IGM result in more significant GO terms, which are closely associated with particular subtypes of gastric cancer according to the previous literature. This demonstrates both the value of including incomplete genes and the ability of IGM to better reproduce the cancer related genes and the corresponding GO terms that have been validated by the previous literature.

## Conclusion

Meta-analysis has been widely used for identifying a more robust set of differentially-expressed genes by integrating multiple microarray datasets. However, some genes with missing replicates, which we referred to as *incomplete genes*, were neglected in previous studies. These genes may also be biologically significant though their statistical significance is not confirmed by all studies. In this paper, we developed Incomplete Gene Meta-analysis for incorporating incomplete genes into the meta-analysis. We have shown that the gene rankings generated by IGM were able to identify more statistically significant genes from incomplete genes in terms of FDR, indicating the benefit of including the incomplete genes. We also applied our algorithm and the traditional methods to three gastric cancer datasets. The over-represented GO terms in each set of significant genes implied that the subsets generated by IGM contained more genes that were associated with the important GO terms relevant to particular clinical annotations in both the breast cancer and gastric cancer datasets. Taken together, these results indicate the benefit in analysing the incomplete genes in addition to complete genes, and demonstrate that IGM is able to appropriately estimate the significance of incomplete genes.

## Authors' contributions

FS, under the supervision of CL and AK, developed the major part of the methodology and ran the major part of the experiments. GA contributed to the Gene Ontology evaluation of the results in the manuscript and the classification evaluation in the Additional File 1. IH contributed to the biological analysis of the results. All authors contributed to the writing and modifications of the manuscript.

## Supplementary Material

Additional file 1 **Supplement**. The supplement contains an analysis of the normality in the five breast cancer datasets, a correlation analysis of the significant genes identified in the five breast cancer datasets, significant Gene Ontology terms in the three gastric cancer datasets and the accuracy of classification in both breast and gastric cancer datasets. Click here for file

Additional file 2 **Source Code**. This additional file contains the source code of the program of our IGM framework, which was implemented using Matlab. In addition, a brief description is included to instruct the use of this Matlab program. Click here for file

Additional file 3 **Ranked GO List**. This additional file contains the full lists of GO terms which are ranked according to their significance in the breast cancer datasets. The GO terms for the gold standard, IGM, INTERSECTION and IGNORE methods are all included in this table. Click here for file
