# Supplementary material for: Meta-analysis of gene expression microarrays with missing replicates
Source: BMC Bioinformatics. 2011 Mar 24;12:84. doi: 10.1186/1471-2105-12-84 (PMC3224118; doi:10.1186/1471-2105-12-84)
Supplement: Additional file 1 — Supplement. The supplement contains an analysis of the normality in the five breast cancer datasets, a correlation analysis of the significant genes identified in the five breast cancer datasets, significant Gene Ontology terms in the three gastric cancer datasets and the accuracy of classification in both breast and gastric cancer datasets. [file 1471-2105-12-84-S1.PDF]

Meta-analysis of gene expression microarrays with missing replicates
Additional File 1 – Supplement

Fan Shi*[1,2], Gad Abraham[1,2], Christopher Leckie[1,2], Izhak Haviv[3] , Adam Kowalczyk[2]

[1]National ICT Australia, Victoria Research Laboratory, Level 2, Building 193, The University of Melbourne Victoria 3010, Australia
[2]Department of Computer Science and Software Engineering, The University of Melbourne, Parkville, Victoria 3010, Australia
[3]Baker IDI Heart and Diabetes Institute, 250 Kooyong Road Caulield, Victoria 3162, Australia

Email: Fan Shi*- shif@csse.unimelb.edu.au; Gad Abraham - gabraham@csse.unimelb.edu.au; Christopher Leckie - caleckie@unimelb.edu.au; Izhak Haviv - Izhak.Haviv@bakeridi.edu.au; Adam Kowalczyk - adam.kowalczyk@nicta.com.au;

*Corresponding author

1 Marginal Normality in Breast Cancer Datasets

In the Methods Section of the paper, we make the assumption that the statistics (Hedges' effect size) for each feature across k datasets follow a k-dimensional normal distribution. A necessary condition of this assumption is that the marginal distribution within each dataset is normal. We have used a normal probability plot to empirically evaluate the approximate normality within each of the breast cancer datasets that we have studied.

For each breast cancer dataset, we computed the Hedges' g effect size as described in the paper for all 22215 probes. The empirical distribution of the statistics was compared to the theoretical normal distribution by quantiles. In Figure 1, we show the normal probability plot for each dataset separately. These figures demonstrate that the statistical values (blue crosses) in each dataset are close to the theoretical normal distribution (red lines).

2 Significant Gene Ontology Terms in the Gastric Cancer Datasets

We list the Gene Ontology terms over-represented in the significant set of genes, which were identified by IGM in the three gastric cancer datasets. Tables 1 and 2 list the significant GO terms (FDR ≤ 0.01) for the genes that are prominently over-expressed in the diffuse and intestinal subtypes, respectively.

Table 1: The 18 GO terms over-represented (FDR ≤ 0.01) in the subset of genes that are prominently over-expressed in the diffuse subtype of gastric cancer. The subsets of genes were generated by the IGM and INTERSECTION methods.

GO ID	P-value	GO Name
GO:0022610	3.71e-012	biological adhesion
GO:0007155	3.71e-012	cell adhesion
GO:0007517	4.47e-007	muscle development
GO:0006936	2.80e-004	muscle contraction
GO:0003012	2.80e-004	muscle system process
GO:0031589	1.33e-003	cell-substrate adhesion
GO:0048731	1.66e-003	system development
GO:0032501	3.01e-003	multicellular organismal process
GO:0030198	3.01e-003	extracellular matrix organization and biogenesis
GO:0007160	3.29e-003	cell-matrix adhesion
GO:0048856	4.20e-003	anatomical structure development
GO:0000902	4.39e-003	cell morphogenesis
GO:0032989	4.39e-003	cellular structure morphogenesis
GO:0007399	4.94e-003	nervous system development
GO:0006928	6.39e-003	cell motility
GO:0051674	6.39e-003	localization of cell
GO:0043062	9.25e-003	extracellular structure organization and biogenesis
GO:0016064	9.75e-003	immunoglobulin mediated immune response

Table 2: 55 GO terms over-represented (FDR ≤ 0.01) in the subset of genes that are prominently over-expressed in the intestinal subtype of gastric cancer. The subsets of genes were generated by the IGM and INTERSECTION methods.

GO ID	P-value	GO Name
GO:0006259	9.70e-011	DNA metabolic process
GO:0051301	1.27e-008	cell division
GO:0007049	3.05e-008	cell cycle
GO:0000278	3.05e-008	mitotic cell cycle
GO:0006260	5.48e-008	DNA replication
GO:0006974	1.51e-007	response to DNA damage stimulus
GO:0006281	1.51e-007	DNA repair
GO:0007067	2.02e-007	mitosis
GO:0000087	2.97e-007	M phase of mitotic cell cycle
GO:0000279	4.68e-007	M phase
GO:0022403	1.73e-006	cell cycle phase
GO:0016126	2.94e-006	sterol biosynthetic process
GO:0022402	1.88e-005	cell cycle process
GO:0007059	1.89e-005	chromosome segregation
GO:0009719	4.99e-005	response to endogenous stimulus

GO:0006334	8.58e-005	nucleosome assembly
GO:0043283	1.17e-004	biopolymer metabolic process
GO:0065004	1.20e-004	protein-DNA complex assembly
GO:0051276	1.50e-004	chromosome organization and biogenesis
GO:0000075	2.11e-004	cell cycle checkpoint
GO:0006139	3.57e-004	nucleobase, nucleoside, nucleotide and nucleic acid metabolic process
GO:0044265	4.87e-004	cellular macromolecule catabolic process
GO:0009113	5.57e-004	purine base biosynthetic process
GO:0007088	5.75e-004	regulation of mitosis
GO:0006695	7.32e-004	cholesterol biosynthetic process
GO:0006066	7.70e-004	alcohol metabolic process
GO:0006007	7.70e-004	glucose catabolic process
GO:0031497	7.71e-004	chromatin assembly
GO:0006694	9.14e-004	steroid biosynthetic process
GO:0006333	1.04e-003	chromatin assembly or disassembly
GO:0046112	1.11e-003	nucleobase biosynthetic process
GO:0008610	1.15e-003	lipid biosynthetic process
GO:0000074	1.25e-003	regulation of progression through cell cycle
GO:0051726	1.34e-003	regulation of cell cycle
GO:0065003	1.64e-003	macromolecular complex assembly
GO:0016125	1.65e-003	sterol metabolic process
GO:0044238	2.22e-003	primary metabolic process
GO:0006096	2.44e-003	glycolysis
GO:0043170	2.85e-003	macromolecule metabolic process
GO:0019320	2.85e-003	hexose catabolic process
GO:0009112	3.03e-003	nucleobase metabolic process
GO:0009057	3.09e-003	macromolecule catabolic process
GO:0046365	3.20e-003	monosaccharide catabolic process
GO:0044275	3.62e-003	cellular carbohydrate catabolic process
GO:0046164	3.67e-003	alcohol catabolic process
GO:0007093	3.67e-003	mitotic cell cycle checkpoint
GO:0007094	3.67e-003	mitotic cell cycle spindle assembly checkpoint
GO:0042987	3.67e-003	amyloid precursor protein catabolic process
GO:0016052	3.79e-003	carbohydrate catabolic process
GO:0022607	3.90e-003	cellular component assembly
GO:0051329	6.85e-003	interphase of mitotic cell cycle
GO:0031577	7.18e-003	spindle checkpoint
GO:0006144	7.18e-003	purine base metabolic process
GO:0051325	9.90e-003	interphase
GO:0006270	9.90e-003	DNA replication initiation

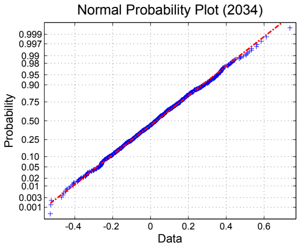

(a) 2034

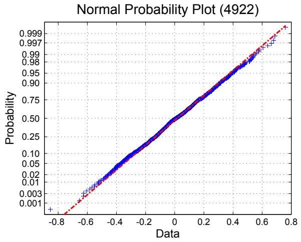

(b) 4922

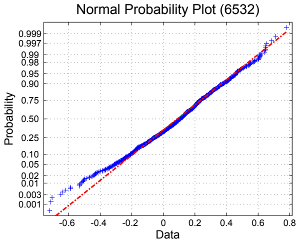

(c) 6532

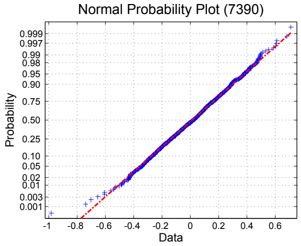

(d) 7390

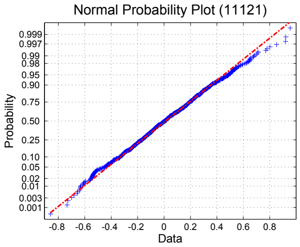

(e) 11121

Figure 1: The normal probability plot for comparing the empirical distribution of the statistics in the breast cancer datasets and the theoretical normal distribution.

3 Accuracy of Classification on Breast Cancer Datasets

In addition to the results presented in Section 3 in the paper, we have also evaluated the effect of using meta-analysis on the accuracy of classification. Our aim is to test how the accuracy of classifier changes when using different numbers of highly ranked features based on their meta-scores.

We evaluated our methods using the Area Under the ROC Curve (AUC) of a centroid classifier [**?**] based on a 5-dataset-fold cross validation of the breast cancer datasets (Section 3.1.4 in the paper). In each fold, a breast cancer dataset was left out (test set), and a meta-analysis method was applied to the other four datasets (training sets) for generating a ranking of all probes. Different numbers of top probes on an exponential scale $(1, 2, 4, \cdots, n)$ were selected according to this ranking to classify the samples in the testing set. Similarly, we obtained the "gold standard" AUC by applying IGM to the original datasets, and evaluated each method by applying it to each group of datasets with simulated missing replicates. The AUC was also averaged across 100 groups of datasets. Since the performance may vary significantly due to the inter-study variation, we show the AUC for each testing dataset separately in Figure 2. The details of the simulation of missing replicates are available in the paper.

It is natural that the "gold standard" outperformed the other meta-analysis methods for small numbers of features since it identified reliable feature rankings based on more information. The IGNORE approach performed worst in the same range because some insignificant incomplete genes were selected due to imprecisely estimated significance, so that the ranking was distorted. We might expect that IGM would also outperform the INTERSECTION method in terms of classification, but the difference between them was not significant. This is demonstrated by the AUC with 95% quantiles across 100 simulations as shown in Figure 2. Note that the quantiles were only computed for the IGM, INTERSECTION and IGNORE methods, since the gold standard corresponded to the original datasets, where there were no missing replicates.

Even though IGM was able to identify more significant genes from the incomplete genes than the INTERSECTION method, the advantage was not necessarily reflected in the AUC of classification. This may be due to two reasons. First, it may be an issue of statistical power — the sample size on which the standard error for the AUC is based may be too small, resulting in wide confidence intervals. Second, the univariate ranking of genes, which by its nature takes into account only the marginal score for each gene, tends to produce a gene list where the top ranked genes are highly correlated. Generally, high (but not perfect) correlation within a set of genes does not necessarily mean that most of them are redundant for purposes of classification [**?**]. However, in this case, it seems that many of the genes assayed are associated with metastasis, and that any subset of the top-ranked genes yields a good classifier. Similar results were reported by [**?**] in their analysis of the [**?**] dataset. Hence, the lists produced by the INTERSECTION are "good enough" and their classification ability is not significantly improved by a better reordering of the genes produced by

IGM. The correlations between the top-ranked genes in this experiment are shown in Section 5 of this Supplement. In addition, our IGM resulted in much smaller variability of the AUC than the IGNORE method, as both methods operated on the union set of features. This observation indicates that IGM estimates the significance of incomplete genes in a more stable way than the IGNORE method. In other words, the IGNORE method is prone to be affected by the changes of missing replicates.

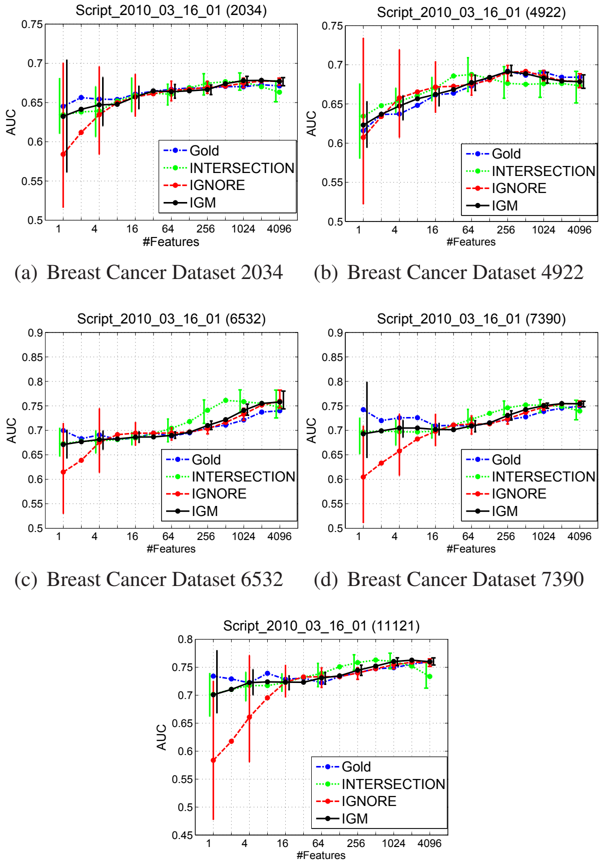

(a) Breast Cancer Dataset 2034 (b) Breast Cancer Dataset 4922

(c) Breast Cancer Dataset 6532 (d) Breast Cancer Dataset 7390

(e) Breast Cancer Dataset 11121

Figure 2: Average AUC and the 95% quantiles across 100 simulations in each breast cancer dataset.

4 Accuracy of Classification on Gastric Cancer Datasets

We also evaluated our methods on three gastric cancer datasets using the AUC of centroid classification based on a 3-dataset-fold cross validation. We have applied IGM, the INTERSECTION method and an approach using Recursive Feature Elimination (labeled "RFE") for feature selection [?] to each pair of gastric cancer datasets (training sets) for generating a ranking of all genes, and this ranking was used to classify the samples in the third dataset (test set). Note

that RFE was also applied on the union set of all genes. Different numbers of the top ranked genes were selected on an exponential scale $(1, 2, 4, \cdots)$ for testing. Since the performance may vary significantly between datasets due to the inter-study variation, we show the AUC for each test dataset separately in Figure 3. The 95% confidence intervals of the AUC are also shown at every position.

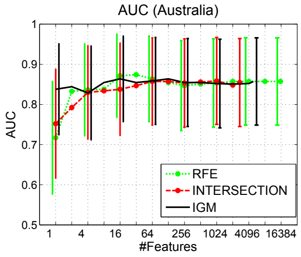

(a) AUC of the Australian dataset

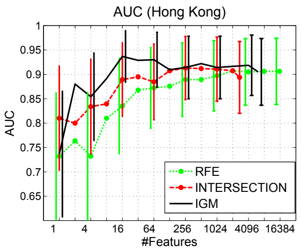

(b) AUC of the Hong Kong dataset

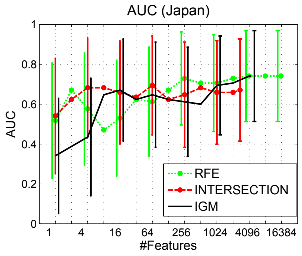

(c) AUC of the Japanese dataset

Figure 3: The AUC of centroid classification conducted on three gastric cancer datasets.

5 Correlation of Highly Ranked Genes

As we described in Section 3 of this Supplement, our IGM approach has the advantage of detecting more significant sets of genes, but does not necessarily result in better accuracy of classification in terms of AUC because of the high correlation between highly ranked genes [?, ?]. To confirm this, we have conducted an exploratory analysis on the five breast cancer datasets to demonstrate the correlation between highly ranked genes.

We first simulated missing replicates for the five breast cancer datasets (100 times) using the method described in Section 3.1.2 in the paper. We then applied IGM to each group of datasets with simulated missing replicates to generate a ranking of all probes, which was identical to the ranking that we have used in the classification evaluation. Based on this ranking, we computed the average correlation coefficient between every pair of probes ranked in the top k $(k = 2^1, 2^2, \cdots, 2^{10})$ in each dataset. Consequently, we had 100 such average correlation coefficients for the 100 simulations. In Figure 4, the distribution of the average correlation coefficients for the top k probes $(k = 2^1, 2^2, \cdots, 2^{10})$ across 100 simulations is shown in a sub-figure for each dataset separately. In Figure 4, we can

observe the consistently high correlation when a small number of genes are selected in every dataset, supporting one of the statement in Section 3 of this Supplement that there is considerable redundancy in the top ranked genes.

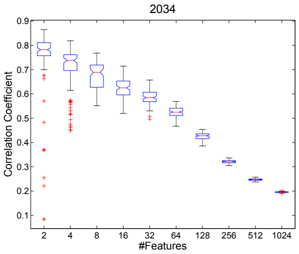

(a) Breast Cancer Dataset 2034

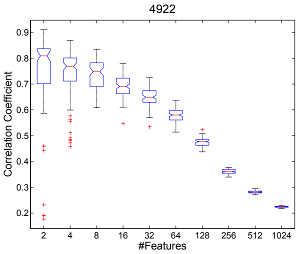

(b) Breast Cancer Dataset 4922

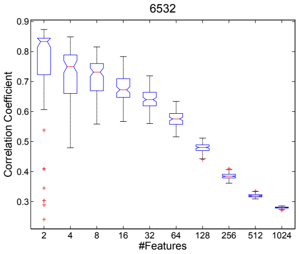

(c) Breast Cancer Dataset 6532

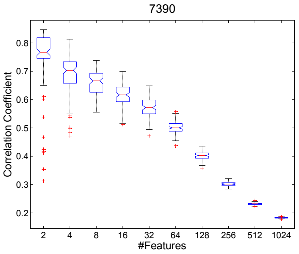

(d) Breast Cancer Dataset 7390

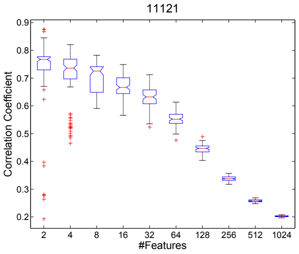

(e) Breast Cancer Dataset 11121

Figure 4: Distribution of the correlation coefficients for the top k genes across 100 simulations in each breast cancer dataset. The horizontal axis is the number of genes selected (k), and the vertical axis is the correlation coefficient.

References
